# Case report: precise mapping and epilepsy surgery of the Broca's area after a comprehensive OPM-MEG investigation of Broca's area localization

**DOI:** 10.1186/s42494-026-00245-7

**Published:** 2026-05-03

**Authors:** Yuanfeng Zhou, Yanjiong Zhang, Luo Tian, Tianshuang Wang, Min Wang, Renqing Zhu, Hao Li, Zhongwei Qiao, Zhigang Cao, Yuming Peng, Ming Ding, Yi Wang

**Affiliations:** 1 Department of Neurology, Children’s Hospital of Fudan University, National Children’s Medical Center, Shanghai, 201102 China; 2 Department of Neurosurgery, Children’s Hospital of Fudan University, National Children’s Medical Center, Shanghai, 201102 China; 3 Department of Radiology, Children’s Hospital of Fudan University, National Children’s Medical Center, Shanghai, 201102 China; 4 Medical Strategic Alliance, Fosun MedTech, Shanghai, 200000 China; 5 School of Instrumentation Science and Opto-Electronics Engineering, Beihang University, Beijing, 100191 China; 6 Magnetoencephalography Joint Laboratory, Children’s Hospital of Fudan University, Shanghai, 201102 China

**Keywords:** OPM-MEG, Broca's region, Localization, Drug-resistant epilepsy

## Abstract

**Background:**

Optically pumped magnetometers magnetoencephalography (OPM-MEG) have demonstrated their value in the diagnosis and mapping of epilepsy, as well as their advantages in pediatric applications.

**Case presentation:**

We present a case of 8-year-old boy with drug-resistant epilepsy, whose epileptogenic lesion is in left Broca’s region. The boy underwent language function evaluation and localization by on-scalp OPM-MEG before surgery. Dipole clusters and Dipole Density of epileptogenic signals by OPM-MEG were located in the left inferior frontal gyrus, though language verbal generation mapping of OPM-MEG signals were mainly located in left frontal orbital gyrus, indicating a localization of the language function area. Seizure freedom and no loss of language function were achieved after MRI-guided laser interstitial thermal therapy.

**Conclusions:**

This article underscores the feasibility of using OPM-MEG to record abnormal discharges of seizure and assess language function area in children, especially in drug-resistant epilepsy surgery involving brain functional areas.

## Background

Epileptogenic lesion is the most common structural cause of drug-resistant epilepsy (DRE) in children. Lesions located in the language-specific area, including Broca’s area (BA44/45) or Wernicke’s area (BA22/39/40), require precise functional mapping in order to avoid the impact of surgical intervention on language function [1]. The available approaches to map the eloquent cortical functions before radical surgery, including functional magnetic resonance imaging (fMRI), Wada test and magnetoencephalography (MEG) [2, 3]. However, fMRI and Wada test are difficult to perform and poorly tolerated in pediatrics, especially in young children with ongoing seizures.

Superconducting quantum interference device magnetoencephalography (SQUID-MEG) was developed in 1969, which has emerged as a valuable tool to assist in the localization of source focus and functional mapping for DRE [3]. However, SQUID-based sensors require one-size-fits-all (typically adult-sized) helmets housing hundreds of sensors requiring cryogenic cooling within liquid helium. Recently, optically pumped magnetometers magnetoencephalography (OPM-MEG) have demonstrated their value in the diagnosis and mapping of epilepsy [4, 5] as well as their advantages in pediatric applications.

## Case presentation

An 8-year-old, right-handed boy first experienced seizures at 5 years and 9 months of age. The seizures manifested as bi-hand-clapping movements with repeated blinking, followed by asymmetric tonicity in the upper limbs and right deviation, accompanied by impaired awareness. Prior to the seizures, he sometimes complained an aura of numbness in his head and body. Subsequently, the seizures became increasingly frequent, occurring more than ten times per day, both during wakefulness and sleep. The initial brain MRI revealed abnormal signals in the left inferior frontal gyrus (IFG), and video-electroencephalogram (VEEG) also showed spike and spike-complexes in the left frontal inferior region. He was diagnosed with focal epilepsy, and was prescribed with oxcarbazepine, levetiracetam, lacosamide, clonazepam, and perampanel. The seizures gradually decreased and were controlled, with the longest period of seizure freedom for 4 months. At 6 years and 5 months of age, he experienced recurrent seizures characterized by repeated blinking with preserved awareness and a same aura, followed by sighing. The seizures occurred in clusters, lasting for 1-2 days, with 1 to more than 10 seizures per day. He was diagnosed with DRE and underwent pre-surgical evaluation. 

**Fig. 1:**
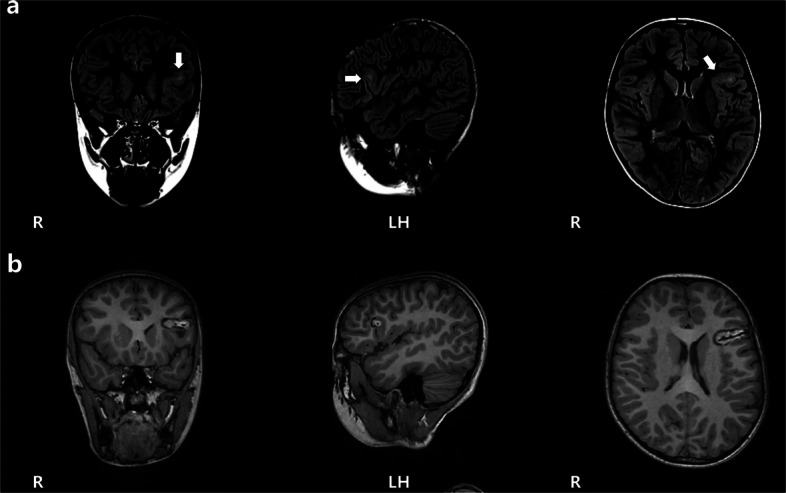
**a** shows abnormal signals in the opercular part of the left IFG on the coronal, sagittal and axial view of MRI. **b** shows the majority of the epileptogenic focus had been destroyed

The patient was the first and only child (G1P1) born at full term via spontaneous vaginal delivery, with a birth weight of 2850g and no history of resuscitation due to asphyxia. His motor and language development milestones were comparable to those of normal peers, and there was no family history of epilepsy. The MRI (T2 Flair 3mm slice) revealed abnormal signals in the opercular part of the left IFG (Fig. 1a). PET demonstrated hypometabolism in the opercular part of the left IFG. VEEG monitoring detected focal seizures originating from the left frontal region. The trio whole-exome sequencing did not identify any explanatory pathogenic variants. The IQ score on the Wechsler Intelligence Scale was 119. After discussion by a multidisciplinary team specializing in refractory epilepsy, it was determined that the epileptogenic focus in the opercular part of the left IFG should be resected or thermally coagulated.

**Fig. 2:**
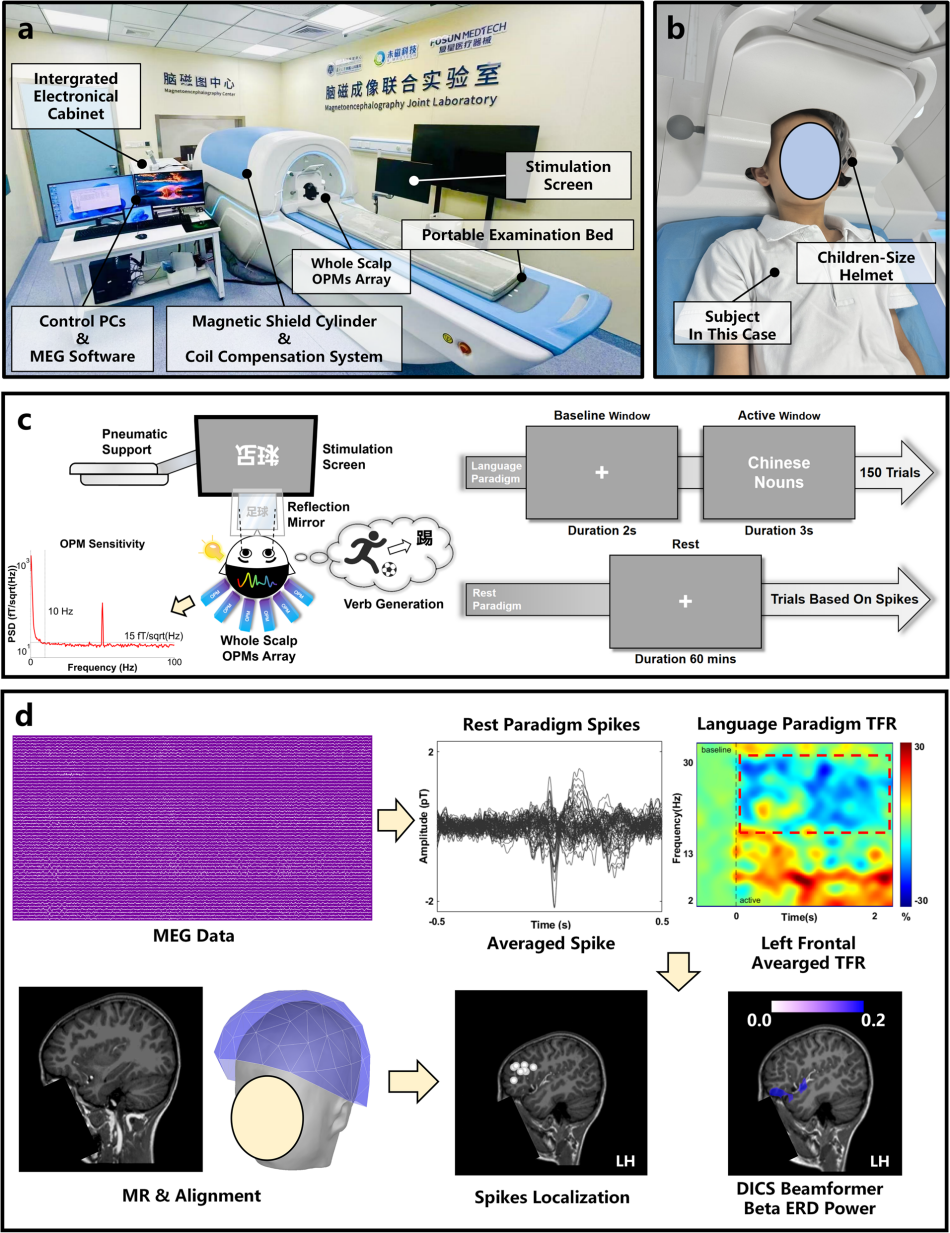
**a** shows the OPM-MEG system in Children’s Hospital of Fudan University. **b** depicts the scenario in which the patient participant from this case study is undergoing an examination. **c** illustrates the paradigm design of the resting-state paradigm and the verb association paradigm used in this case study. It also presents the specific process of verb association: the participant views the stimulus screen through a reflection mirror and associates verbs with the Chinese nouns displayed on the screen, e.g., when the noun "football" is displayed, the associated verb would be "kick". **d** Briefly outlines the data processing workflow for the MEG paradigm. MEG data and MR data are co-registered and integrated. For resting-state data, dipole fitting is performed based on the marked epileptiform spikes (20 dipoles were been analyzed). For language-related regions, the beta event-related desynchronization (ERD) signals observed in the left frontal lobe of the participant are source-localized using DICS Beamformer to map the energy distribution

However, focal seizures originated from left IFG, which was considered as the classical Broca’s area (BA44/45), the resection of the epileptic focus may impact motor language function. Wada test and fMRI was refused by the patient because of the invasiveness of Wada test and poor tolerance to the MRI examination due to the loud noise. The localization of the dominant language functional areas was detected by an 128-channel OPM-MEG system (Marvel MEG 128, Beijing X-Magtech Medical Technologies Limited, China) with whole scalped covered OPM arrays consisted of 128 channels fixed to a children-size helmet, with a magnetic shielding system that spares huge expense in construction of a magnetic shielding room (Fig. 2a&b). All OPM sensors’ empty room power spectrum density ensure the level remains below 15fT/sqrt (Hz) between 10–100Hz frequency band.

The participant in this case underwent two OPM-MEG examinations: The first one was a resting-state examination which the purpose was to detect epileptiform discharges. The patient rested in a supine position with eyes closed, remaining body relaxed and keep his head as still as possible. The examination lasted a total of 60 minutes. The second was a language task-state examination [6]. A verb-association paradigm was used, with each trial consisting of a 2-s baseline resting state followed by a 3-s presentation of a Chinese noun (Fig. 2c). A total of 150 verb generation trials were repeated.

The MEG data sequentially underwent synthetic gradiometer [7], filtering and ICA steps [8], which respectively remove far-field interference, power frequency interference, and heartbeat and ocular artifacts. In the sensor space phase, resting-state data were reviewed by three MEG technicians to identify and mark epileptiform discharges. For language task data, time-frequency representations were computed using the multitaper method [9] and baseline-corrected. A singleshell model was computed as the forward model [10]. Finally, arrays’ coordinates were aligned to participants’ native MR space and spatial mapping was performed: dipole fitting was used for epileptiform discharges (Fig. 2d), while a DICS beamformer [11] was applied to the language task data to demonstrate the localization of the power decline contrast between the beta band during verb generation and the resting state, with only results above 70% of the maximum value was displayed.

**Fig. 3:**
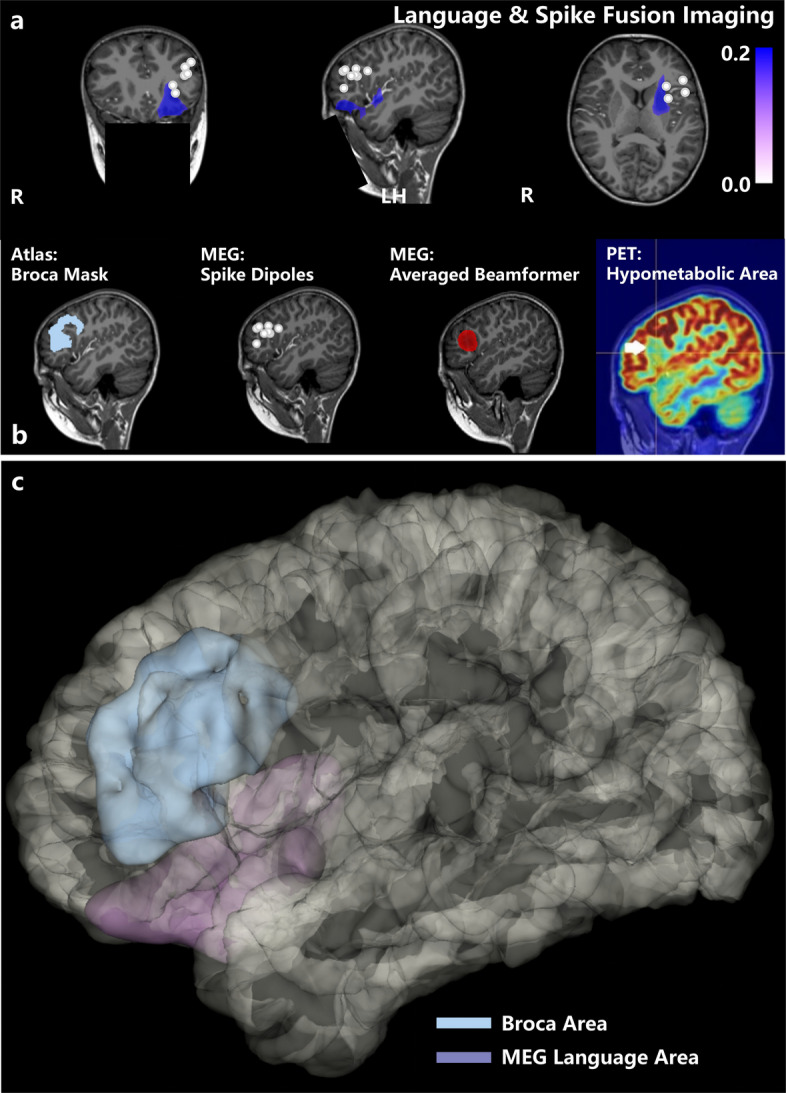
**a** overlays the dipole localizations with the language beamformer results, where the language paradigm localization demonstrates a predominant left-hemisphere dominance and high-signal areas include the left orbitofrontal cortex, insula and superior temporal gyrus. The overlap between the two datasets is minimal and the language distribution significantly differs from the classical Broca's area. **b** shows the sagittal slice of the subject's left hemisphere language area. From left to right, the first column represents the Brodmann area Broca region mask, the second column displays the epileptic discharge region localized by MEG, the third column displays the MEG spikes averaged beamformer results and the fourth column shows the hypometabolic region identified by PET-CT. The spatial locations of these four regions are highly consistent. **c** presents the differences between the subject's Broca area and the MEG-based language localization in the surface space. The MEG threshold is set to 60% of the maximum, and no overlap is observed between the two regions

The dipole localization analysis revealed the participant's epileptiform spikes are primarily located in the middle frontal gyrus, inferior frontal gyrus, and insular cortex, while the language paradigm localization demonstrates a predominant left-hemisphere dominance and high-signal areas include the left orbitofrontal cortex, insula and superior temporal gyrus (Fig. 3). The overlap between the two datasets is minimal and the language distribution significantly differs from the classical Broca's area. Due to the patient's young age, given that the patient was unable to cooperate with functional MRI or Wada test, the OPM-MEG findings were the only quantitative data available to localize functional language area.

The epileptogenic focus underwent MRI-guided Laser Interstitial Thermal Therapy (MRgLITT). A follow-up MRI showed that the majority of the epileptogenic focus had been destroyed (Fig. 1b). Post-surgery, there were no impairments in language comprehension or expression. After 6 month follow-up, the seizures were controlled and no language dysfunction occurred.

## Discussion

Historically, classical Broca’s area (BA44 and BA45) was associated with language processing in the brain, surgery of Broca’s area is often avoided to preserve language function [12]. fMRI was recommended for lateralizing language functions in place of Wada test in temporal epilepsy or extratemporal epilepsy patients (level C) [13]. In this pediatric case, the epileptogenic focus was confirmed to be located in the opercular part of the left IFG by dipole source localization using OPM-MEG. However, the actual motor language function area was confirmed to localize in left-hemisphere dominance by OPM-MEG, including the left orbitofrontal cortex, insula and superior temporal gyrus, with no overlap between the classical and actual Broca’s area. Although Wada and fMRI examination were absent because of the tolerance in this case, seizure freedom and no loss of language function were achieved after MRgLITT of epileptogenic lesion, reconfirming the localization of Broca's region.

Numerous previous studies have demonstrated the localization value of SQUID-MEG in identifying the motor and language dominant hemispheres [6, 14]. However, SQUID-MEG requires a helmet of a fixed size that must be cryogenically cooled with liquid nitrogen, posing limitations, especially in pediatric applications. In recent years, OPM-MEG, as a non-invasive functional neuroimaging technique, has garnered increasing attention, with its application in functional mapping and surgical planning for pediatric epilepsy theoretically [15]. In this case, the OPM-MEG, with 128-channels fixed to a children-size helmet, with a magnetic shielding system without magnetic shielding room, can provide satisfactory spatial resolution for precise localization of language function before surgery.

## Conclusions

This case underscores the feasibility of using OPM-MEG to record abnormal discharges of seizure and assess language function area in children, thereby paving the way for future clinical trial to evaluate the diagnostic value of OPM-MEG in the pediatric epilepsy population, especially in drug-resistant epilepsy surgery involving brain functional areas.

## Data Availability

The datasets used and analyzed during the current study are available from the corresponding author upon reasonable request.

## Abbreviations

OPM-MEG: Optically pumped magnetometers magnetoencephalography
SQUID-MEG: Superconducting quantum interference device magnetoencephalography
DRE: Drug-resistant epilepsy
fMRI: Functional magnetic resonance imaging
IFG: Inferior frontal gyrus
VEEG: Video-electroencephalogram
MRgLITT: MRI-guided Laser Interstitial Thermal Therapy
